# 

**Published:** 1887-03

**Authors:** 


### BOOK NOTICES.

CELESTIAL SONGS.—This is the title of a collection of songs and hymns by B. M. Lawrence, M. D. The volume contains 128 pp. and nearly that number of songs, with the written music, about the fourth of which are original. In looking over the collection, we find a number of old acquaintances, plainly recognizable in their new dress, and the absence of sectarian refrains set to doleful music, which sound like a chorus of despairing groans, is a feature we can but highly commend.

NOVEL METHODS OF TREATING DISEASES OF THE MIDDLE EAR, by Seth S. Bishop, M. D., Chicago. This is a treatise read by its author, who is Attending Surgeon to the Illinois Charitable Eye and Ear Infirmary, etc., etc., at the annual meeting of the Illinois State Medical Society. The system of treatment herein described is claimed to be, not only new, but so simple as to be available to the afflicted without the intervention of a surgeon, which makes it all the more valuable.

"THE THERAPEUTICAL DRINKING OF HOT WATER—ITS ORIGIN AND USE," is the title of a treatise by Ephraim Cutter, M. D., New York, which goes fully into the merits and methods of this simple curative agent, and also the particulars of "a new mode of alimentation" in chronic diseases, known as the "Salisbury Plans," which cannot fail to serve as a useful manual in the hands of hospital nurses, who are also gifted with heads, and are willing to learn how to use both, in ministering to the sick. Published by Kellogg, 9 West 29th St., New York City.

THE DIETETIC ANNUAL FOR 1887, issued by WELLS & RICHARDSON CO., Burlington, Vt., as a Manual of Health alone, is a work of great value, not only to the invalid, but to those who value good health, and seek the surest means of preserving it. The prepared *Lactated Food* of this company, suited to the tenderest infant, as well as the adult of all ages and conditions, has acquired a deservedly wide popularity and correspondingly extensive use in families, in nurseries and hospitals, as shown by the ample testimonials contained in this handy little manual, wherein the fullest details are given for its use in various cases. We are assured that this food contains no harmful ingredients—nothing indeed to benumb the organs of sensation, and prey upon delicately-organized nervous systems. On the contrary, we are assured that it is altogether nutritious and upbuilding, and, as such, should rapidly displace those injurious compounds with which the drug stores are so plentifully supplied, to the detriment of good health.

BUCHANAN'S JOURNAL OF MAN.—The first number of this monthly publication, revived by Prof. Joseph Rodes Buchanan, now of Boston, is before us. Dr. Buchanan's name has been so intimately associated with the foremost moral, educational, medical and political reforms which have agitated the public mind for the last half-century, that the mention of it in connection with the foregoing publication, under the old-time name, will doubtless draw to it an extensive patronage. The Salutatory is a ringing declaration of independence of mummified creeds and systems founded in superstition and sustained by the influences of priestcraft and the offerings of unreasoning credulity. Its promises are to expose error, uphold truth, and assist struggling humanity to higher levels of thought and activity. Long life and prosperity to this new worker in a field where the needs are many and the laborers few, and poorly enough sustained.

THE CENTURY MAGAZINE, for March, continues its interesting biography of Lincoln, bringing it down to the period of the repeal of the Missouri Compromise. The war article is a paper on Secretary Stanton, whose photograph forms that frontispiece. Other good articles are two on Faith-healing, discussing both sides of the question. The White Man of the South, French Sculptuary and Art in our Coinage.

This Magazine still continues the foremost of our monthlies and sustains the high standard it has set for itself.

WE have received No. 1, of Vol. 7, of *Fort Wayne Journal C Medical Science*, an excellent publication, which we gladly welcome to our exchange list.

